# High school science fair: Student opinions regarding whether participation should be required or optional and why

**DOI:** 10.1371/journal.pone.0202320

**Published:** 2018-08-10

**Authors:** Frederick Grinnell, Simon Dalley, Karen Shepherd, Joan Reisch

**Affiliations:** 1 Department of Cell Biology,UT Southwestern Medical Center, Dallas, Texas, United States of America; 2 Department of Physics, Southern Methodist University, Dallas, Texas, United States of America; 3 Plano Independent School District, Plano, Texas, United States of America; 4 Department of Clinical Sciences, UT Southwestern Medical Center, Dallas, Texas, United States of America; Medical College of Wisconsin - Central Wisconsin Campus, UNITED STATES

## Abstract

The goal of our research is to identify strengths and weaknesses of high school level science fair and improvements that might enhance learning outcomes based on empirical assessment of student experiences. We use the web-based data collection program REDCap to implement anonymous and voluntary surveys about science fair experiences with two independent groups—high school students who recently competed in the Dallas Regional Science and Engineering Fair and post high school students (undergraduates, 1st year medical students, and 1st year biomedical graduate students) on STEM education tracks doing research at UT Southwestern Medical Center. Herein, we report quantitative and qualitative data showing student opinions about the value of science fair. Few students in any group thought that competitive science fair (C-SF) should be required. The most common reasons given for not requiring C-SF were no enjoyment and no interest in competing. On the other hand, student attitudes towards requiring non-competitive science fair (NC-SF) were nuanced and ranged as high as 91%, increasing with student maturation, science fair experience, and STEM track. The most common reasons given for requiring NC-SF were learning scientific thinking skills and research skills. Students opposed to requiring NC-SF most frequently mentioned no enjoyment and no interest in science. Several student comments critical of the fairness of science fair led us to determine possible differences in science fair experiences depending on whether or not students received help from scientists. Those who received help from scientists had an easier time getting their research idea, more access to articles in books and magazines, and less difficulty getting resources. We discuss the idea that two different types of science fairs—competitive science fair with a performance goal orientation and non-competitive science fair with a mastery goal orientation—might be required to promote the broad goal of educating all students about science and engineering.

## Introduction

Next Generation Science Standards (NGSS) describes practice of science as one of three key dimensions of science education [1]. How best to introduce this experience into the science curriculum lacks widespread consensus. One possibility is science fair. Science fair brings together many of the elements of practice—problem selection, experimental design and implementation, data analysis, and communication of research findings–and by doing so offers students one of the few opportunities in science education to experience for themselves these practices combined.

Science fair has a long history and is widely implemented as part of informal and formal science education across schools in the United States [2] although reported by some to be on the decline [3,4]. The significance of national and international science fair competitions was recognized in President Obama’s 2011 State of the Union Address, *We need to teach our kids that it’s not just the winner of the Super Bowl who deserves to be celebrated*, *but the winner of the science fair* [5]. And at the 2018 Sundance Film Festival, the film *Science Fair* won the Festival Favorite Award. Yet despite the visibility and attention, few empirical studies have been published that examine the high school science fair experience and its impact. Most of the science fair literature consists of anecdotal essays, both favorable [6–9] and unfavorable [10–12].

Several years ago, we began a research project using an anonymous and voluntary survey to study students’ high school science fair experiences. Our goal was to identify strengths and weaknesses and improvements that might enhance STEM learning outcomes. Since science fairs across the U.S. occur in many different formats, a small, regional survey might have been useful locally but without having broader implications. To aim for a more robust view of science fair and to learn if students shared similar experiences notwithstanding different structures of science fairs, we studied two independent groups: a local group of high school students who recently had participated in the Dallas regional science fair and a state/national group of students who were post high school and at different degrees of advancement along STEM-relevant educational paths. In the Dallas regional fair, approximately one thousand middle school and high school students present projects that are judged by community volunteers (professional scientists and engineers). Results of scoring determine awarding of 1st-2nd-3rd prizes in different categories as well as grand prizes and special awards from scientific organizations, and winning students often go on to compete in state and national science fairs.

Our previously published survey findings focused on questions we asked students about their overall science fair experiences. We observed similarity and many common features of the high school fair experience reported by the high school and post high school students regarding sources of help, types of help received, obstacles encountered, and ways of overcoming obstacles [13]. In the same surveys, we also asked questions focused on student opinions regarding the value of science fair. We asked the quantitative question: *Do you think science fair should be required or optional*? and the qualitative, open-ended text question: *Reason Why*? And we asked students these questions for both competitive and non-competitive science fair. Students’ answers to these questions are the main subject of the current report. By including the option of comparing competitive vs. non-competitive science fair, we hoped to learn student attitudes towards competition. By comparing high school vs. post high school student groups, we hoped to learn if maturation of students’ views about high school science fair occurred as they pursued their STEM education tracks.

Overall, we found that very few students thought competitive science fair (C-SF) should be required. The most common reasons given for not requiring C-SF were no enjoyment and no interest in competing. On the other hand, student attitudes towards requiring non-competitive science fair (NC-SF) were much more nuanced and ranged as high as 91%, increasing with student maturation, science fair experience, and STEM track. The most common reasons given for requiring NC-SF were to learn scientific thinking skills and research skills. In response to several student written comments about the fairness of science fair, we also determined possible differences in science fair experiences depending on whether or not students received help from scientists. Those who received help from scientists had an easier time getting their research ideas, more access to articles in books and magazines, and less difficulty finding resources. Based on our findings, we discuss the idea that two different types of science fairs, competitive and non-competitive, might be required to promote the broad goal of educating all students about science and engineering.

## Materials and methods

This study was approved by the UT Southwestern Medical Center (UTSW) IRB–Study #STU 072014–076. Study design entailed administering to students a voluntary and anonymous online survey as described previously [13]. Briefly, surveys were carried out using the REDCap survey and data management tool [14]. Survey content, the same as in our previous report [13], was adapted from an earlier study by Montreal sociologists [15] and included questions about student demographics, type of science fair participation, help expected and received, obstacles encountered, and solutions implemented to overcome obstacles. The high school and post high school surveys can be found in supporting information (S1 Survey and S2 Survey, respectively). Two pairs of the survey questions were the primary focus of the current paper–one quantitative: *Do you think science fair projects should be optional or required*? And the other qualitative and open-ended: *Reason why*? We asked these two questions about both non-competitive and competitive science fairs.

Four different student groups participated in the surveys. One group consisted of high school students (grades 9–12) who recently had competed in the Dallas Regional Science and Engineering Fair (DRSEF). The students were mostly from a suburban Dallas district with a strong commitment to science education that encourages but does not require students to carry out a science fair project. The other three groups were post high school students in one of the research programs at UTSW: undergraduates doing summer research as part of the Summer Undergraduate Research Fellow (SURF) program; 1st year graduate students in the biomedical sciences; and 1st year medical students doing a summer research project.

The complete survey data set can be found in supporting information (S1 Dataset). Quantitative data were analyzed by frequency counts. Percentages were tabulated and sorted to compare different answer selections. Data shown in the figures are presented graphically to make overall trends easy to appreciate. We also show the data in tables beneath the graphs to show the actual numbers. Significance of answers was determined using Chi-square contingency tables for independent groups or McNemar Chi-square analysis for paired responses of a single group. Where p values 0.05 or lower were determined, significance is indicated in the graphs and in the tables including the p values.

Qualitative text analysis was accomplished using an approach modeled on NVivo (http://www.qsrinternational.com/). Initially, two members of the research team (FG and SD) independently coded 80 (about 15%) of the student comments why science fair should be optional or required and categorized these comments into a preliminary matrix of shared student reasons (nodes). After merging the findings, the preliminary matrix was the starting point to analyze the entire set of 570 student comments, which were independently re-coded, and the starting matrix was revised so as to be representative of the entire collection of student answers. Two cycles of independent re-coding and revision achieved final harmonization of the *Reason Why* categories based on 280 student comments about competitive science fair and 290 student comments about non-competitive science fair. Longer student comments sometimes expressed more than one idea, in which case, the comments were coded into more than one *Reason Why* category. The complete set of student answers to the *Reason Why* question and corresponding reason category assignments can be found in supporting information (S2 Dataset).

## Results

### Demographics

Table 1 summarizes information about the students who participated in the survey. The student groups are the same as described previously [13] except for an additional graduate student cohort surveyed in November 2016 and one less high school student whose original data turned out to be duplicated. The first and second rows show that that response rate to the survey was close to 60% for high school students but less for the post high school groups. Based on the number of students who completed surveys, 100% of the high school students and ~23% of the post high school students competed in science fair. Almost all students who completed surveys answered the two quantitative questions regarding whether non-competitive and competitive science fairs should be optional or required, and a high percentage answered the two qualitative, opened ended *Reason Why* text questions. Of the students who reported that they had competed in science fair, <10% of the high school students and ~40% of the post high school students had been required to do so. (The high school students were mostly from a suburban Dallas district with a strong commitment to science education that encourages but does not require students to carry out a science fair project.)

**Table 1:** Survey demographics.

Group	High school students	Post high school student group			
		All	Undergrad	Med	Grad
# Students sent surveys	112	713	324	196	193
# (%) Students who completed surveys	64 (57.1)	267 (37.4)	148 (48.7)	62 (31.6)	57 (29.5)
**Responses of students who completed the science fair survey**					
# (%) Participated in science fair	64 (100)	62 (23.2)	38 (25.7)	13 (21.0)	11 (19.3)
# (%) Answered quantitative questions about required or optional science fair	64 (100)	263 (98.5)	145 (98.0)	62 (100)	56 (98.2)
# (%) Answered *reason why* questions about required or optional science fair	63 (98.4)	227 (86.3)	132 (91.0)	52 (83.9)	44 (78.6)
# (%) Required to compete in science fair (based on those who actually participated)	4 (6.3)	25 (40.3)	13 (34.2)	8 (61.5)	4 (36.4)

### Should science fair be optional or required—Quantitative findings

**Fig 1:**
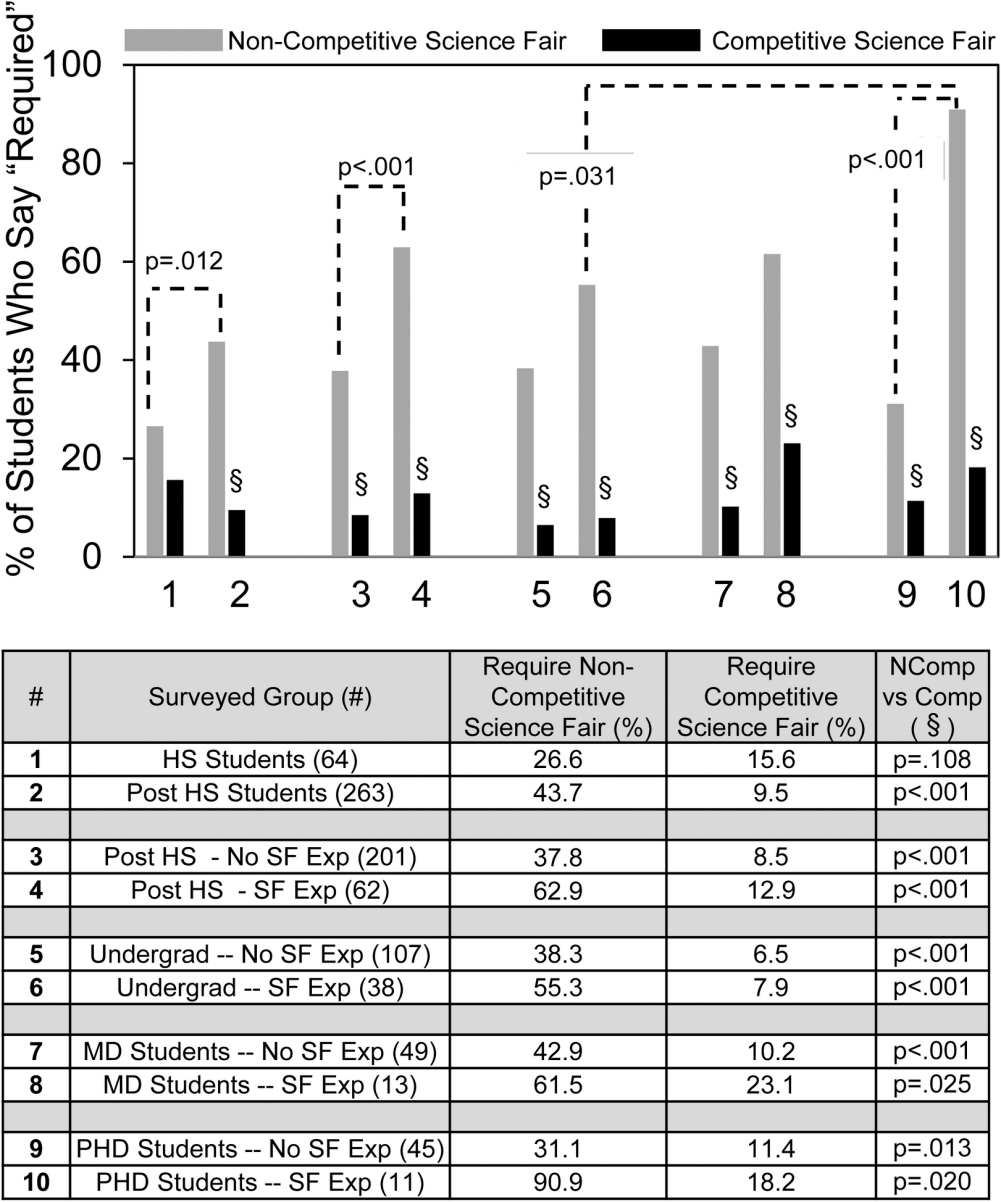
Student answers to the question “Should science fair be optional or required?”

Fig 1 shows the numbers and percentages of students who checked “required” as the answer to the following questions: (i) *Do you think science fair projects should be optional or required*? *(This need not be for science fair competition*.*)*; (ii) *Do you think science fair projects for competition should be optional or required*? The grey bars show the % of students who checked non-competitive science fair should be required; the black bars show the % of students who checked competitive science fair should be required.

Survey answers depend on question wording and interpretation. We did not know how the students would interpret the idea of non-competitive science fair. By including non-competitive science fair, we hoped to learn student attitudes towards competition. Given the qualitative results (discussed later), the students appeared to interpret the difference between competitive vs. non-competitive question in a straightforward manner that permitted them to express their attitudes towards competition.

Few students (~1 in 5 or less) thought that competitive science fair (C-SF) should be required (black bars). For every group, more students thought that non-competitive science fair (NC-SF) should be required (gray bars) compared to C-SF. For instance, row #1 shows that of the 64 high school students, 26.6% favored requiring NC-SF vs. 15.6% favored requiring C-SF. Except for row #1, the preference of every student group for requiring NC-SF vs. C-SF reached statistical significance (§) with p values shown in the far right column of the table.

Fig 1 organizes the findings to show a comparison of student responses according to different groups. We observed differences depending on student maturity, science fair experience, and STEM track. Post high school students were more likely to require NC-SF than high school students (#2 vs. #1). (Where the p values for the independent group comparisons reached significance, they are shown in the graphs by brackets and not in the tables.) Post high school students with SF experience were more likely to require NC-SF than post high school students without SF experience (#4 vs. #3). Subdividing the post high school groups into undergrad, MD and PHD students groups showed a similar pattern. That is, students with SF experience were more likely to require NC-SF than students without SF experience (#6 vs #5, #8 vs #7; #10 vs #9), but the findings only reached statistical significance for the PHD students. Finally, the percentage of PHD students with SF experience who checked requiring NC-SF (~90%) was highest overall.

### Should science fair be optional or required—*Reason Why* categories

Analysis of the almost 600 student answers to the *Reason Why* question (280 comments about competitive science fair and 290 comments about non-competitive science fair) showed that the students were thoughtful in their responses and provided useful narrative data. In reading the comments we observed commonly expressed reasons and sometimes more than one reason in the same comment. We organized the reasons into 14 categories (7 favorable and 7 unfavorable) regarding why science fair should be optional or required. Doing so still captured much of the richness of the student comments and was necessary to compare the qualitative data with the quantitative data and to learn if student attitudes changed according to maturity, science fair experience, and STEM track.

Table 2 shows the favorable and unfavorable *Reason Why* categories and the total number of times each was mentioned for non-competitive (NC) and competitive (C) science fair. Categories called *Other favorable* and *Other unfavorable* included diverse reasons mentioned at low frequency that did not fall elsewhere. Overall, students offered 200 reasons favorable vs. 215 unfavorable to require non-competitive science fair and 73 reasons favorable vs. 321 unfavorable to require competitive science fair. Because student comments could express more than one reason (see below), the total number of reasons expressed (415 for NC-SF and 394 for C-SF) exceeded the total number of student comments.

**Table 2:** Reason Why categories and examples from student comments explaining why non-competitive (NC) and competitive (C) science fair should be optional or required.

Reason Category	# Times Cited		Examples—When a single comment is used to express more than one reason, the text relevant to the idea is underlined.
	NC	C	
**Reasons Favorable to Require Science Fair**			
Scientific Thinking Skills	72	7	Thinking and planning as a scientist is an important skill in life. Also, expressing your ideas in a manner that is understandable to others is critical in almost any field of study. These are all skills that are tested and honed by conducting science projects.
Research Skills	32	5	It's a great introduction to research, which is difficult to come by in high school.
Communication and/or Presentation Skills	19	15	Thinking and planning as a scientist is an important skill in life. Also, expressing your ideas in a manner that is understandable to others is critical in almost any field of study. These are all skills that are tested and honed by conducting science projects.
Intro to Scientific Knowledge	35	1	1) You get to explore different aspects of science 2) Put your knowledge into practical applications
Career Interests	14	2	I think it's a great way to encourage student's to think creatively about science. Most student's loose the sense of exploration during high school due to standardized testing. This allows them to experience it at a time when deciding college degrees and career path.
Competition Incentive	0	43	The competition element of the science fair project adds an incentive to do well and encourages students to take personal responsibility for their project.
Other Favorable	28	0	Those not interested with science fair or science in general should not be required to complete a project. However, optional projects are very beneficial to students who are interested in science.
**Reasons Unfavorable to Require Science Fair**			
Not everyone interested in science	78	43	Those not interested with science fair or science in general should not be required to complete a project. However, optional projects are very beneficial to students who are interested in science.
No enjoyment; overall negative attitude	63	76	People that don't want to do them will approach the project with a bad attitude and take away from those who actually care about it.
Negative behaviors and future consequences	22	25	People that don't want to do them will approach the project with a bad attitude and take away from those who actually care about it.
Not enough time or money	33	38	It takes a lot of time, so requiring competition level science projects is too time consuming and may be difficult for some students.
Don't Want/Like to Compete	0	72	Some students don't like the idea of competition and are solely interested in sharing knowledge.
Winning vs. Learning	3	39	I don't think the point of a science fair is to win a competition. It should be about learning about a topic
Other Unfavorable	16	28	Projects are of higher quality when optional. I love science but I don't think it should be forced on anyone

Table 2 also shows examples of student comments. The complete set and corresponding reason category assignments can be found in supporting information (S2 Dataset). Sometimes a comment expressed more than one type of reason. In the examples in the table, if a single comment could be placed into more than one reason category, then the text relevant to the category is underlined. For instance, the student comment—*Thinking and planning as a scientist is an important skill in life*. *Also*, *expressing your ideas in a manner that is understandable to others is critical in almost any field of study*. *These are all skills that are tested and honed by conducting science projects*.*–*was placed into both the *Scientific thinking skills* and *Communication and/or presentation skills* categories. The student comment—*People that don’t want to do them will approach the project with a bad attitude and take away from those who actually care about it*.–was placed into both the *No enjoyment; overall negative attitude* and *Negative behaviors and future consequences* categories.

### Should science fair be optional or required—Qualitative findings

**Fig 2:**
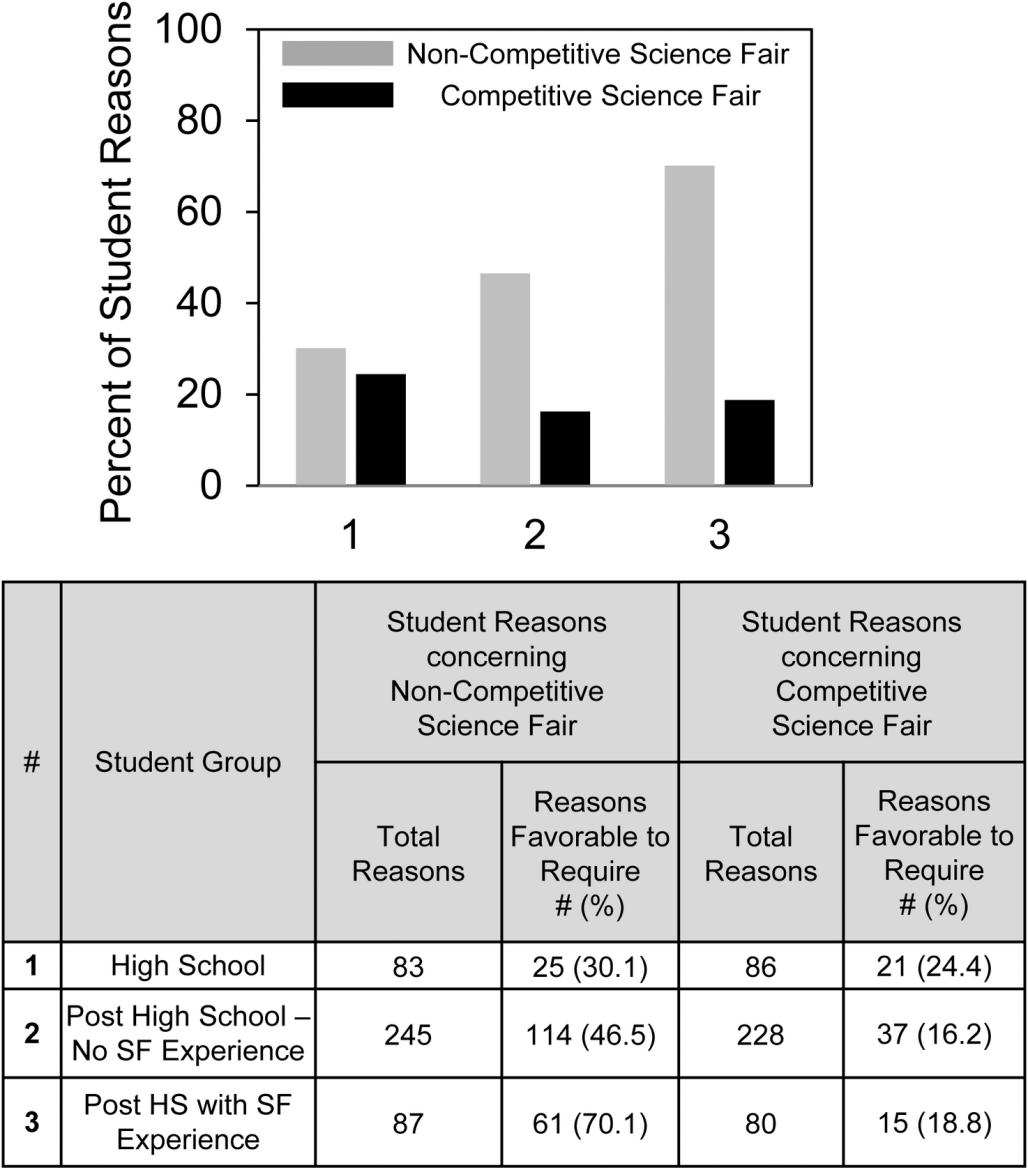
Frequency of student reasons why non-competitive and competitive science fair should be required.

Fig 2 summarizes the numbers and percentages of student reasons to require non-competitive science fair (grey bars) and competitive science fair (black bars). These data are based on qualitative counts of reasons. The findings based on the qualitative data (student comments) resembled the quantitative findings (yes/no answers) shown in Fig 1. Consistent with the quantitative findings, regardless of age or science fair experience, students expressed fewer reasons to require C-SF (black bars) compared to NC-SF (gray bars). Also, the frequency of favorable reasons towards requiring NC-SF increased with student maturity and science fair experience. That is, post high school students without science fair experience expressed more positive reasons than high school students (47% vs. 30%), and post high school students with science fair experience expressed more positive reasons than post high school students without (70% vs. 47%).

**Fig 3:**
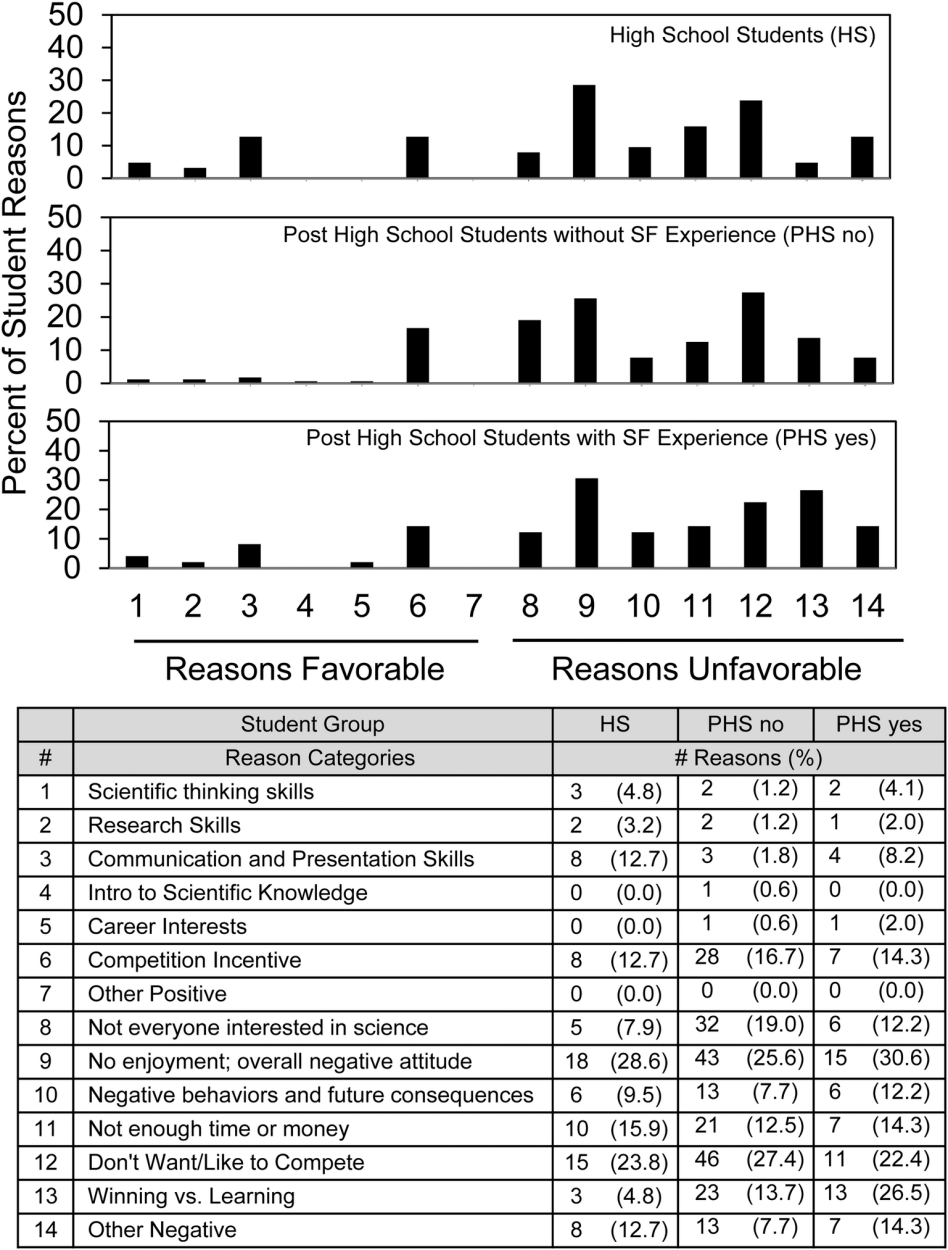
Distribution of reasons favorable and unfavorable to require competitive science fair.

**Fig 4:**
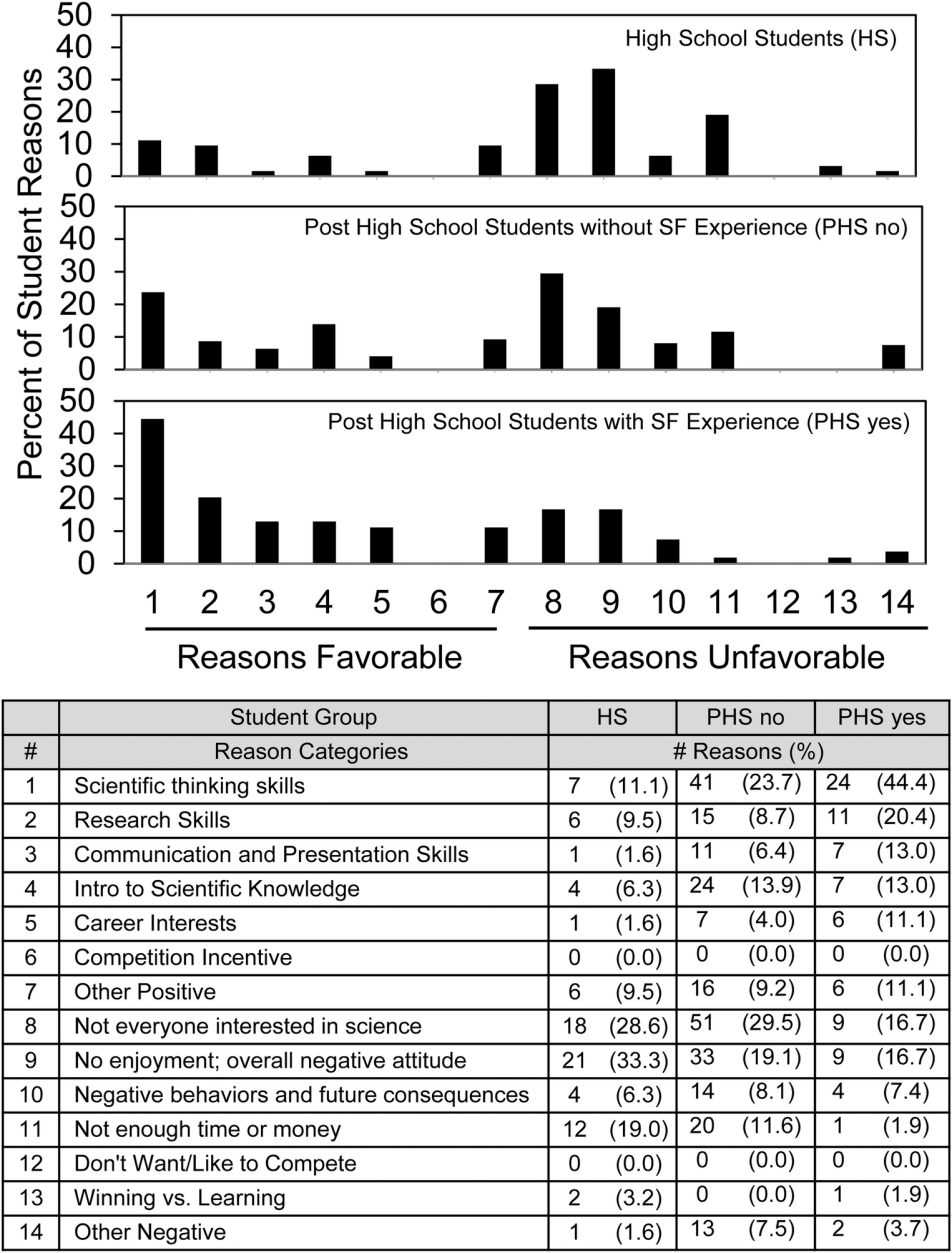
Distribution of reasons favorable and unfavorable to require non-competitive science fair.

To take further advantage of the narrative responses, Fig 3 (C-SF) and Fig 4 (NC-SF) show the number and per cent of students’ reasons favorable or unfavorable to require science fair and parse the data according to whether the students were in high school, post high school without science fair experience, and post high school with science fair experience. Regarding competitive science fair (Fig 3), the overall pattern showed little difference between the three groups. The most frequently expressed reasons not to require science fair were equally split between *No enjoyment/overall negative attitude* (#9) and *Don’t want/like to compete* (#12). Especially post high school students did not favor requiring competitive science fair because it emphasized *Winning vs*. *learning* (#13). *Competition incentive* (#6) was the only favorable reason common to all three groups.

Regarding non-competitive science fair (Fig 4), the pattern of reasons showed a distinct shift depending on student maturity and science fair experience. The reasons favorable to require science fair given most frequently by the post high school students with science fair experience were *Scientific thinking skills* (#1, 44% of the reasons) and *Research skills* (#2, 20% of the reasons). By contrast, these two reasons were rarely mentioned by high school students. On the negative side, *No enjoyment/overall negative attitude* (#9) continued to be expressed frequently now combined with *Not everyone interested in science* (#8). High school students, more so than others, also mentioned *Not enough time or money* (#11) as an unfavorable reason.

### Differences in science fair experience according to whether or not students received help from scientists

In our previous analysis of the science fair experience survey, we did not compare possible differences in student science fair experience depending on whether or not the students received help from scientists [13]. However, in reading the students *Reason Why* comments, we noticed several students expressed concerns about the fairness of science fair. One wrote: *On the national level*, *science fairs are dominated by students whose parents have PhDs and connections to place them in university labs… The obstacles for a student who does not have those connections are large and it is unfair that the current science fair environment is so rigged to award the privileged*. Another wrote: *The ceiling of the project mainly depends on how well you are connected to a researcher at a higher institution*. *Many participants had family members or good connections for them to work in their lab*. *As a result*, *the playing field felt unfair to those who were not well connected in science or who had families who didn't have scientific backgrounds*. Given these comments, we evaluated further the larger science fair survey data set.

**Fig 5:**
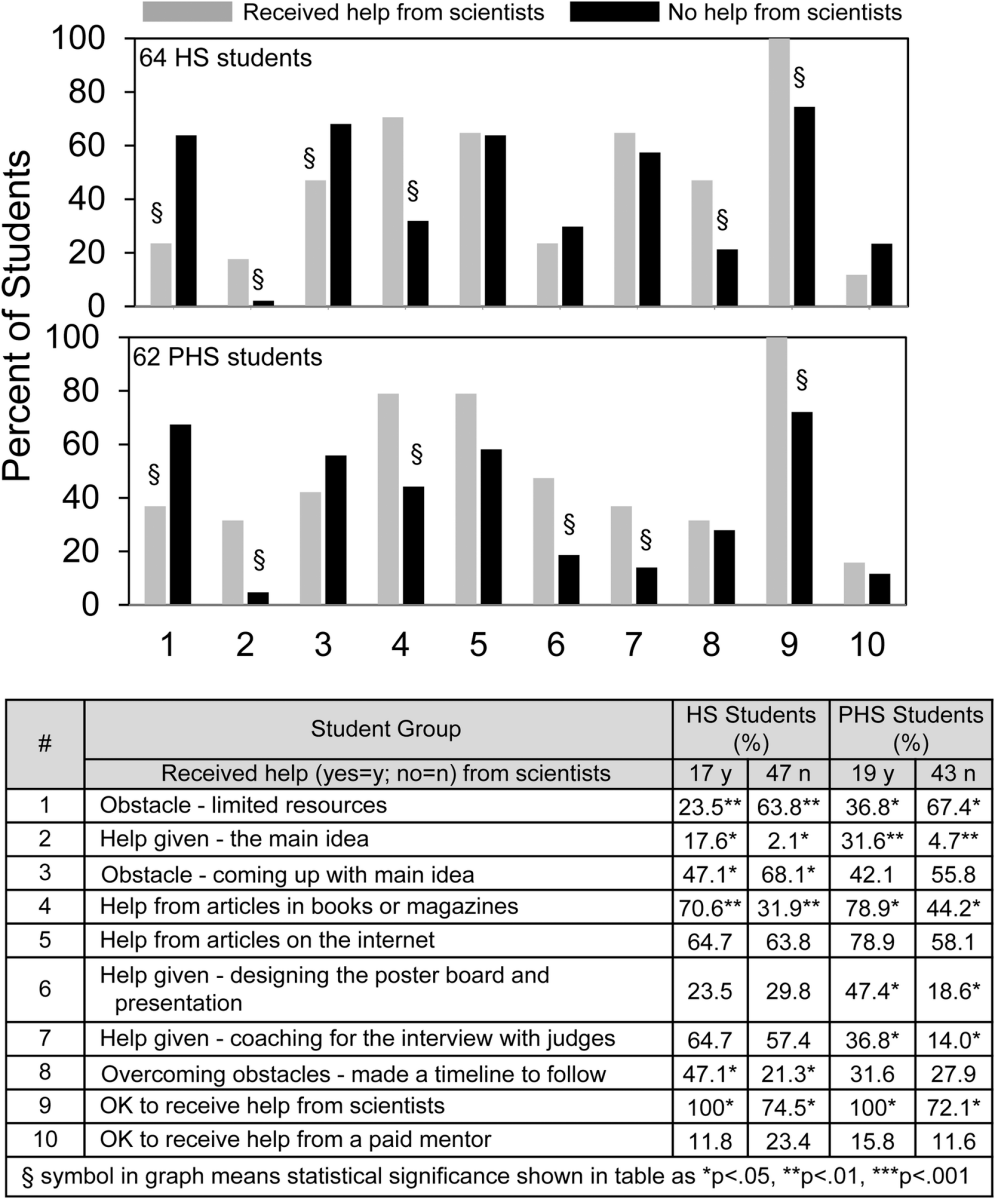
Different science fair experiences of high school (HS) and post high school (PHS) students depending upon whether or not they received help from scientists.

The survey permits students to choose up to 10 possible sources of help, 11 possible types of help, 12 possible obstacles, and 15 possible ways to overcome obstacles. Of these 48 items, 7 showed significant differences in student science fair experience depending on whether or not the students reported receiving help from scientists. Fig 5 summarizes these differences and several related findings. Students who received help from scientists were less likely to experience limited resources as an obstacle (#1). They were more likely to be given their idea (#2) and less likely to experience coming up with the idea as an obstacle (#3). They were more likely to get help from articles in books or magazines (#4), whereas students with and without help from scientists were equally likely to get help from articles on the internet (#5). Other differences observed were designing the poster board (#6), coaching for the interview (#7), and making a timeline to follow (#8), but these latter three differences were not common to both high school and post high school student groups. (Comparisons reaching statistical significance are shown by § in the graphs with p value ranges in the table.)

Fig 5 also shows that among students who did not receive help from scientists, 72–75% still checked that receiving help from scientists was reasonable (#9). On the other hand, regardless of whether or not they received help from scientists, < 25% of the students thought that receiving help from a paid mentor was reasonable (#10). Notwithstanding the benefits of receiving help from scientists, Table 3 shows that having that help did not show any consistent effect on student attitudes towards requiring non-competitive or competitive science fair.

**Table 3:** Different science fair experiences of high school (HS) and post high school (PHS) students depending upon whether or not they received help from scientists.

Student Group	HS Students (%)		PHS Students (%)	
Received help (yes = y; no = n) from scientists	17 y	47 n	19 y	43 n
Think that non-competitive science fair should be required	35.3	23.4	47.4	69.8
Think that competitive science fair should be required	5.9	19.1	21.1	9.3

## Discussion

The ongoing goal of our research is to identify strengths and weaknesses of high school level science fair and improvements that might enhance learning outcomes based on empirical assessment of student experiences reported in anonymous and voluntary surveys. Since science fair across the U.S. occurs in many different formats, we studied two independent student groups to gain a broad perspective of science fair experience notwithstanding possible different structures of science fairs: a local group of high school students who recently had participated in the Dallas regional science fair and a state/national group of students who were post high school and at different degrees of advancement along STEM-relevant educational paths doing research at UT Southwestern Medical School. Previously, we described similarity and many shared features of high school fair experience between these two student groups regarding sources of help, types of help received, obstacles encountered, and ways of overcoming obstacles [13].

In the current report, we present data about the students’ opinions regarding the value of science fair. We asked students the quantitative question whether science fair should be optional or required and the qualitative open ended text question *Reason why*. The students provided thoughtful written answers to the qualitative question, which was an indication that they answered the survey questions seriously. The findings based on assessment of the qualitative data (student comments) closely resembled the quantitative findings (yes/no answers) lending further support to the validity of the survey findings.

Regarding competitive science fair, few high school and post high school students selected “required” (~10–20%). Based on the qualitative findings, the most common reasons given for not requiring competitive science fair were *no enjoyment/overall negative attitude* (26–31%) and *Not everyone likes to compete* (22–27%), followed by *Not enough time or money* (13–16%) and *Not everyone interested in science* (8–19%). The only positive thing high school and post high school students wrote about competitive science fair, albeit to a lesser extent, was *Competition incentive*. Of particular significance, less than 5% of the students in any of the groups mentioned *Scientific thinking skills* and *Research skills* as possible favorable reasons to make competitive science fair required.

The overall negative attitude of the students towards requiring competitive high school science fair could not be explained by personal experience since only 40% of the post high school and 6% of the high school students were required to do science fair. Moreover, the post high school students who did not participate in science fair were just as negative about requiring science fair as those who did participate. In short, what students appeared to dislike was the idea of being required to compete. And the post high school students with science fair experience made the insightful suggestion that competition focused science fair experience more about *Winning vs*. *learning* (27%).

Student responses regarding whether non-competitive science fair should be required were more nuanced. High school students still were negative overall but more positive compared to their view of competitive science fair (27 to 16%). Almost half of the post high school students were favorable towards requiring non-competitive science fair, which increased to 63% for post high school students with high school science fair experience and 91% of the post high school PHD students with science fair experience. Analysis of the *Reason Why* question showed a marked shift in the features on which students focused. Post high school students with science fair experience mentioned *scientific thinking skills* and *research skills* frequently (44% and 20% of reasons) up from <5% of the reasons given requiring competitive science fair. Also, many more students mentioned *Communication and presentation skills*, *Introduction to scientific knowledge*, and *Career interests* as favorable to require non-competitive science fair. The most important negative aspects were *No interest in science*, *No enjoyment*, and *Not enough time/money*, and these were mentioned less frequently.

Two features may combine to explain the differences in student attitudes towards requiring competitive and non-competitive science fair. First, the highly negative attitude of students regarding forced competition may simply undermine consideration of all other factors. Second, maturation along STEM educational paths may allow the more advanced students to appreciate retrospectively the potential and actual benefits of doing science fair. One limitation worthwhile noting about the high school student responses is that although these students were not required to participate in science fair, we don’t know if they actually were interested in science. Whether or not the high school students were interested in science might affect their opinions about requiring science fair. That is a question that we hope to answer in future studies.

The view of many post high school students that non-competitive science fair should be required focuses attention on the importance of promoting different science fair models. In a non-competitive, standards-based approach to science fair, judges (who no longer would need to be outsiders) could assess on a sliding scale student progress towards mastery of the different practices of science, just those practices emphasized by NGSS [16]. The idea has been discussed in the science fair literature but only for younger students [6,17]. Ironically, at the time science fair began in New York City around 1930 as a derivative of after-school science clubs [2], students in the science clubs were engaged in both competitive and non-competitive activities centered around the use of science toys. On the competitive side, toy manufacturers offered cash prizes to winning entries describing original uses of the products that would be of general interest to others and not simply variations of already published ideas. On the non-competitive side, club members could win prizes by accumulating points for individual achievements such as constructing an apparatus, demonstrating an apparatus, and performing an experiment, etc. [18].

A standards-based, non-competitive science fair would fit the National Science Teachers Association educational objectives that participation in science competitions should emphasize learning experience rather than competition and a focus on scientific process, content, and/or application [19]. Non-competitive science fair also would represent an approach consistent with research on student motivation described by goal orientation theory. Analysis of the impact of classroom climate on student motivation has led to the conclusion that mastery orientation (i.e., competition with oneself with emphasis on understanding and improving skills and knowledge) provides more effective learning motivation compared to performance orientation (i.e., competing with others with emphasis on demonstrating high ability and grades) [20,21].

Factors contributing to success in competitive science fair include parental support and encouragement [22], social and research resources [23], and access to outside of school facilities [24]. Because of the availability of such factors to only a subset of students, the criticism sometimes has been made in the literature that science fair is fundamentally unfair [10,25,26]. In their *Reason Why* comments, several students stated the view that competitive science fair was unfair because students who do the best get help from parents who are scientists or have science connections. We had not analyzed previously the ways in which science fair experience differed depending on whether or not students received help from scientists [13]. We observed three important differences. With help from scientists, students had an easier time getting their research idea, more access to help from articles in books and magazines, and less difficulty getting the resources to carry out their projects. Getting help from scientists did not influence overall student attitudes towards whether science fair should be optional or required. Nevertheless, the importance of getting help from scientists for student success would be less relevant in the non-competitive science fair framework.

The National Research Council (NRC) Framework underlying NGSS described two aims for STEM education, one oriented to science for everyone and the other oriented to science for the scientists and engineers of the future,

(1) educating *all* students in science and engineering and (2) providing the foundational knowledge for those who will become the scientists, engineers, technologists, and technicians of the future. [27] (italics added)

An important policy implication of our findings worth considering is that two different science fair formats—non-competitive and competitive–might be necessary to promote the broad NRC science education aims. Given its history, competitive science fair with a performance goal orientation likely will continue to be viewed by many as a means to educate the scientists and engineers of the future. However, non-competitive science fair with a mastery goal orientation might be a better approach to help educate all students about science and engineering.

## Supporting information

S1 Dataset Excel dataset showing all of the survey questions and answers described in this paper. (XLSX)

S2 Dataset Excel dataset showing the complete set of reason category assignments. (XLSX)

S1 Survey Survey questions used with DRSEF students. (PDF)

S2 Survey Survey questions used with UTSW graduate/medical students. (PDF)
